# Esophagitis in a Post‐Liver Transplant Patient: A Case of Cytomegalovirus and Herpes Simplex Virus‐1 Coinfection

**DOI:** 10.1002/ccr3.9565

**Published:** 2024-11-19

**Authors:** Ammad Javaid Chaudhary, Taher Jamali, Abdullah Sohail, Christian E. Keller, Allyce Caines, Mazen ELatrache

**Affiliations:** ^1^ Internal Medicine Department Henry Ford Hospital Detroit Michigan USA; ^2^ Department of Gastroenterology and Hepatology Henry Ford Hospital Detroit Michigan USA; ^3^ The University of Iowa Hospitals and Clinics Iowa City Iowa USA

**Keywords:** achalasia, cytomegalovirus, esophagitis, Herpes Simplex Virus‐1, liver transplant

## Abstract

In post‐liver transplant patients, esophagitis presents a diagnostic and management challenge due to the potential for opportunistic infections. This case describes a 59‐year‐old female with primary sclerosing cholangitis who underwent orthotopic liver transplantation six years prior. She presented with dysphagia, and her medical history included immunosuppression with prednisone, tacrolimus, and mycophenolate and a history of achalasia treated with esophageal peroral endoscopic myotomy. Esophagogastroduodenoscopy (EGD) revealed severe esophagitis with extensive ulcerations, raising suspicion for infectious etiologies such as cytomegalovirus (CMV) and herpes simplex virus‐1 (HSV‐1). The biopsy confirmed a rare coinfection of CMV and HSV‐1, which was characterized histologically by viral cytopathic effects and immunohistochemical staining. Treatment with valganciclovir and temporary cessation of mycophenolate led to symptom resolution and viral clearance. Follow‐up EGD demonstrated healing of esophageal ulcers, with subsequent findings of Candida esophagitis but no evidence of CMV or HSV recurrence. This case highlights the importance of early endoscopic evaluation and biopsy in immunocompromised patients with esophagitis. CMV and HSV‐1 coinfection, while rare, should be considered in this population due to its association with severe complications such as perforation and bleeding. Timely antiviral therapy and immunosuppression adjustment are critical for favorable outcomes.

**Summary**

- In post‐liver transplant patients with esophagitis, consider CMV and HSV‐1 coinfection, particularly with severe symptoms; early diagnosis via EGD and biopsy facilitates timely antiviral treatment and immunosuppression adjustment.

## 1 Introduction

This case report discusses the clinical presentation, diagnosis, and management of a rare coinfection of cytomegalovirus (CMV) and herpes simplex virus‐1 (HSV‐1) in a post‐liver transplant patient. It highlights the clinical challenges and implications of managing esophagitis in immunocompromised individuals.

## 2 Case History/Examination

The patient, a 59‐year‐old female with a history of primary sclerosing cholangitis, had undergone orthotopic liver transplantation from a CMV seropositive donor 6 years prior. She presented with a 1‐week history of dysphagia. Her immunosuppression regimen post‐transplant included prednisone, tacrolimus, and mycophenolate. She also had a history of achalasia secondary to scleroderma, which was treated with esophageal peroral endoscopic myotomy (EPOEM) 1 year ago. The differential diagnosis included infectious agents like CMV, HSV, and Candida, typical in immunocompromised patients with esophagitis.

## 3 Methods

**FIGURE 1:**
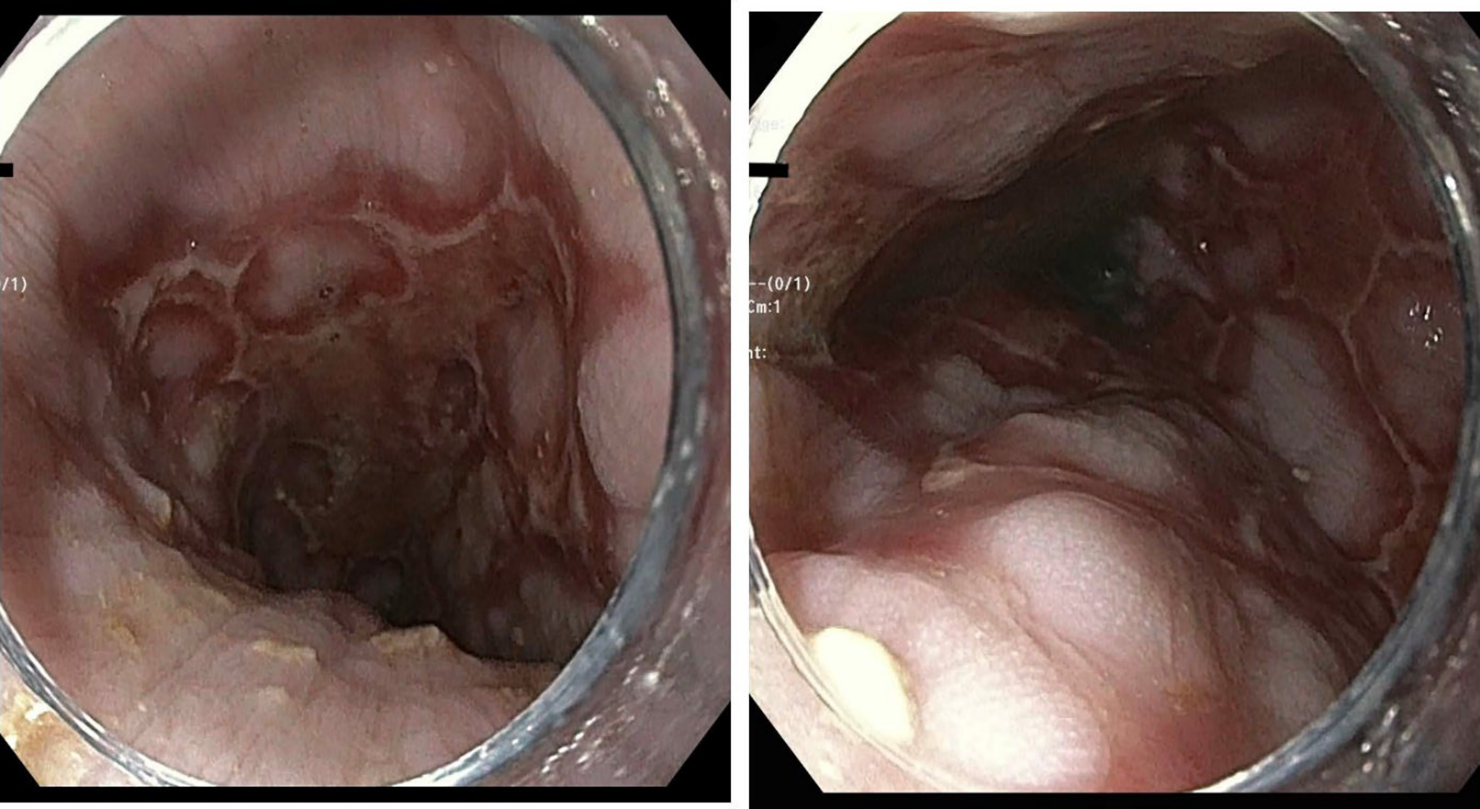
The extensive ulceration observed during EGD.

The diagnosis was confirmed via esophagogastroduodenoscopy (EGD), which revealed severe esophagitis characterized by extensive serpiginous and confluent non‐bleeding ulcerations (Figure 1).

## 4 Results and Conclusion

**FIGURE 2:**
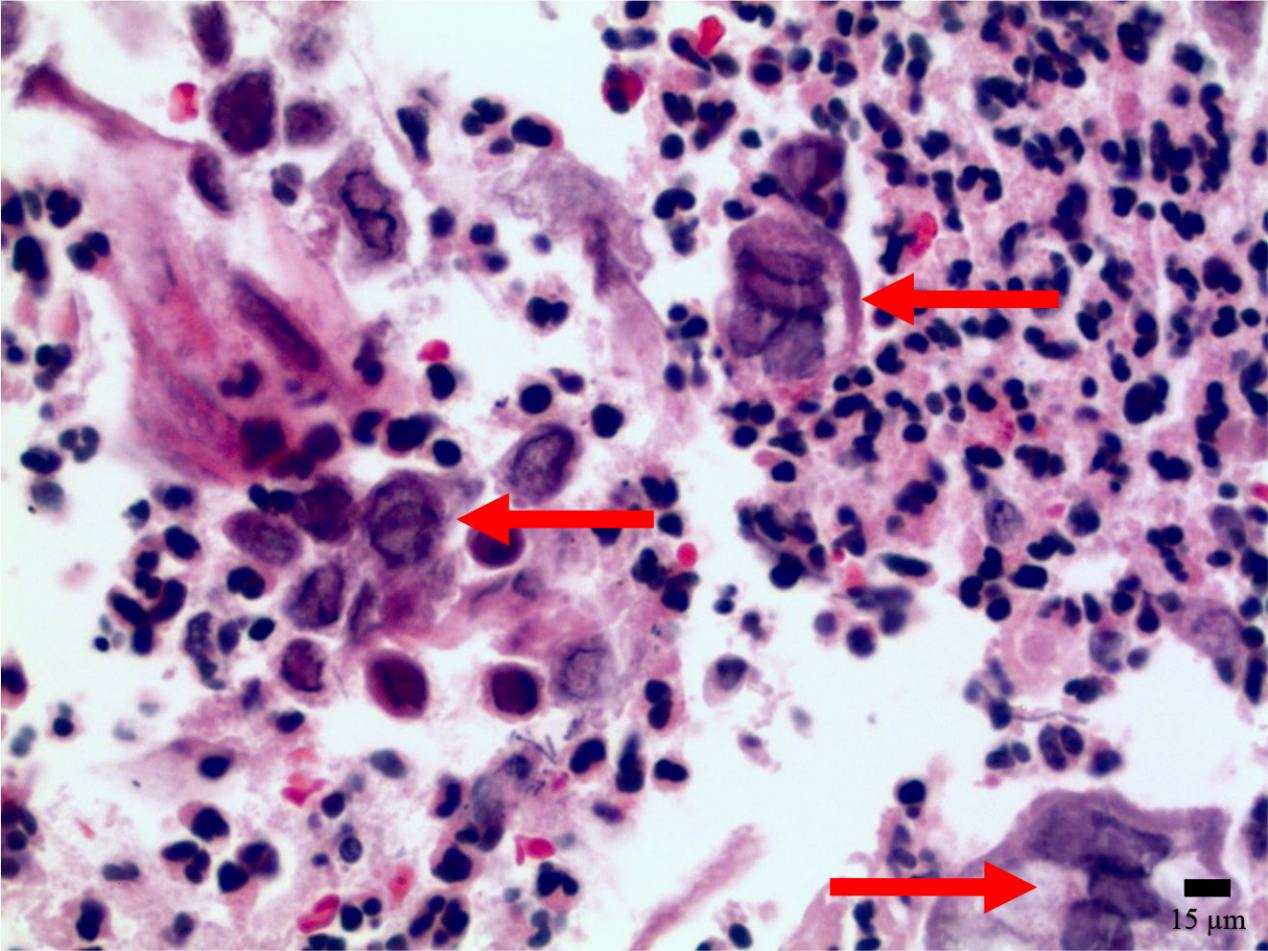
Hematoxylin and eosin‐stained section from the junction of an ulcer base and adjacent esophageal squamous mucosa. There are multinucleate squamous epithelial cells (arrows) with powdery chromatin in the center of the nuclei, nuclear molding, and chromatin margination.

**FIGURE 3:**
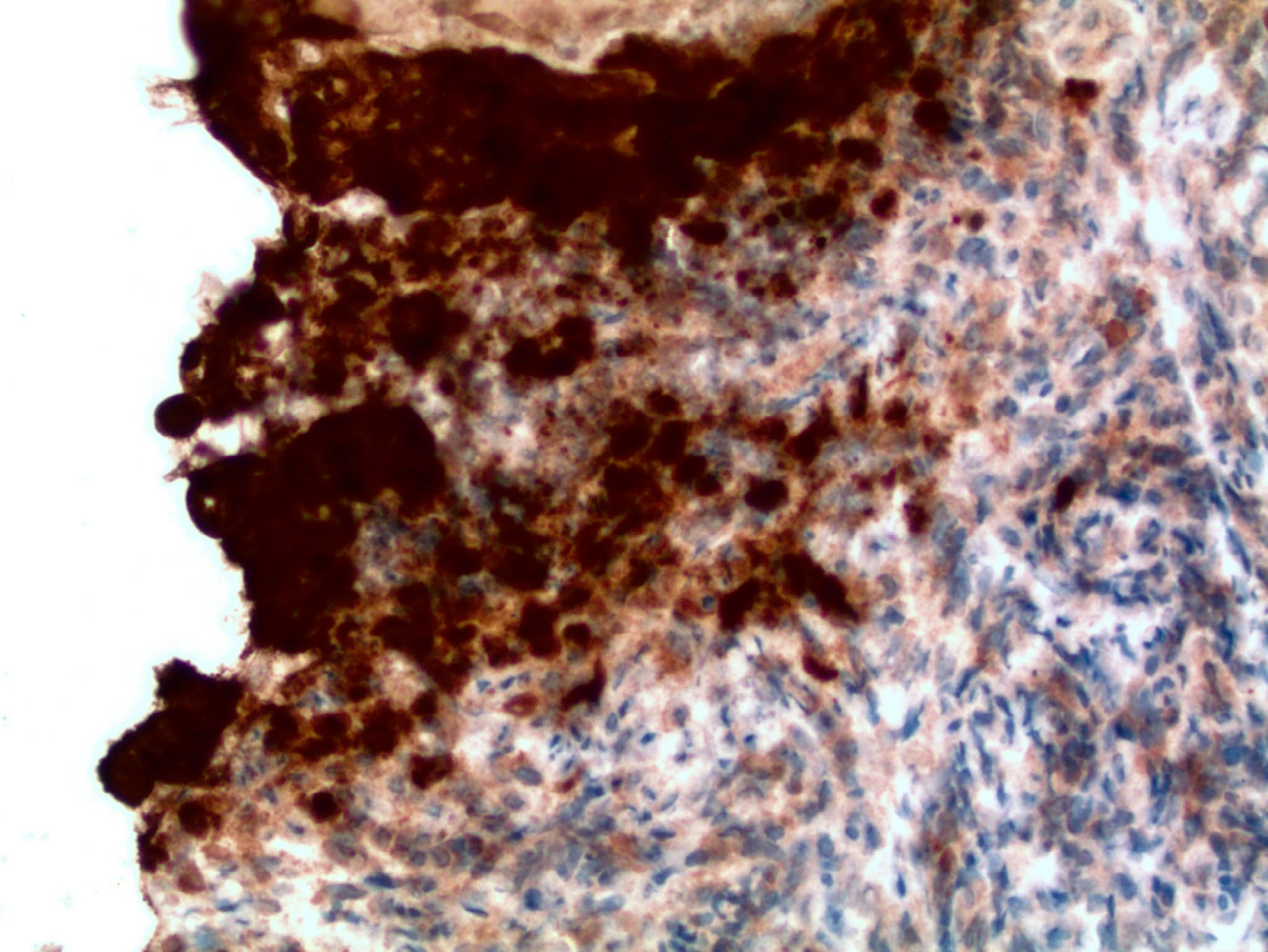
Immunohistochemical stain with antibodies against HSV1 epitope. Brown reaction product highlights squamous epithelial cell nuclei containing viral particles. The positive signal colocalizes with the multinucleate cells at the junction of ulcer base and non‐ulcerated squamous mucosa.

**FIGURE 4:**
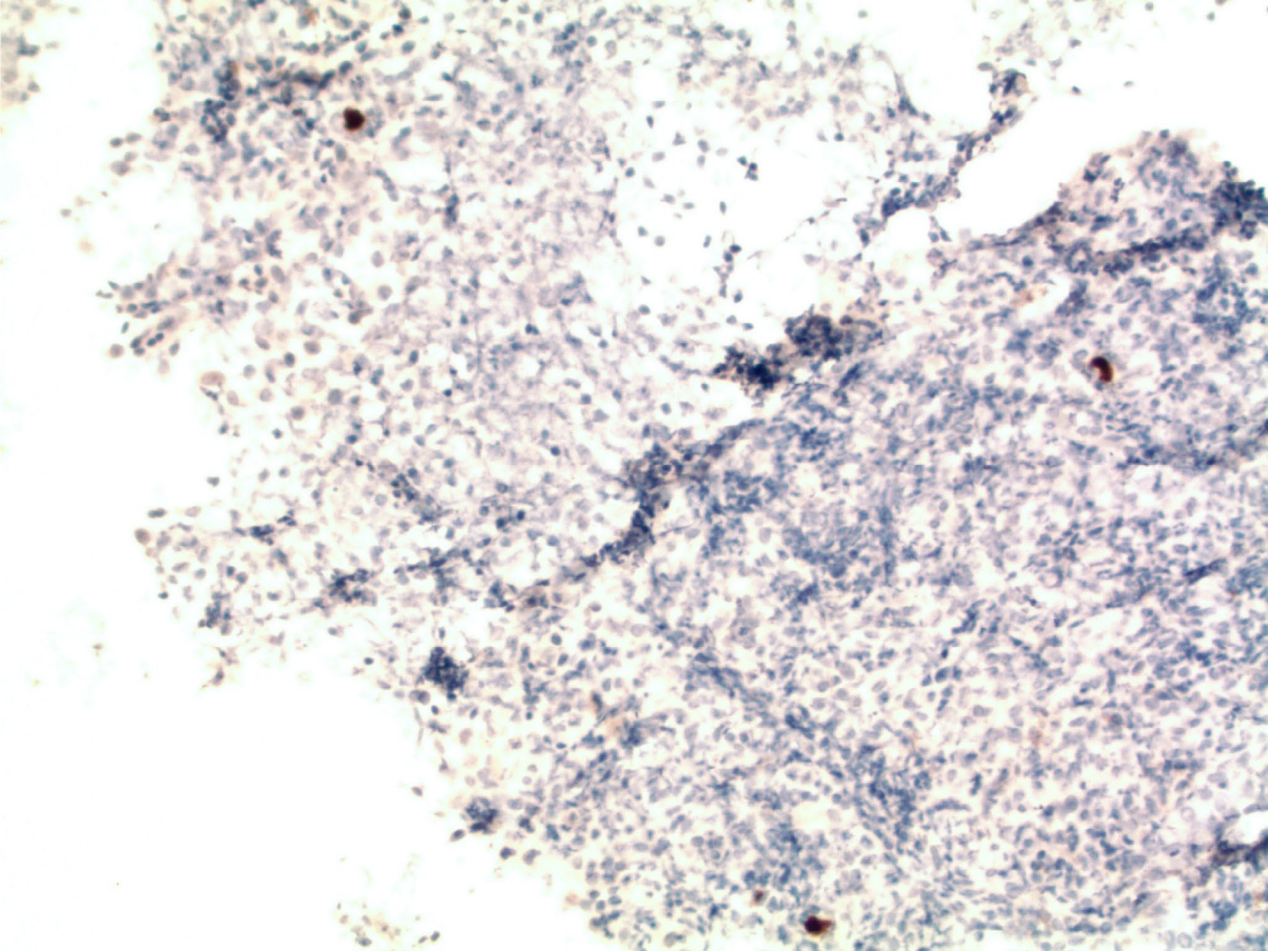
Immunohistochemical stain with antibodies against CMV epitope. Brown reaction product highlights nuclei containing viral particles in the ulcer base. Viral epitopes are predominantly found in endothelial cells.

Biopsies confirmed a coinfection of CMV and HSV‐1 (Figures 2, 3, 4). Treatment commenced with valganciclovir and a temporary halt to mycophenolate, resulting in undetectable CMV levels subsequently. A follow‐up EGD conducted 2 months later showed that the esophageal ulcers had no new bleeding, and biopsies indicated candida esophagitis without signs of CMV or HSV infection. The diagnosis of CMV and HSV‐1 coinfection is a rare occurrence but crucial to identify due to its association with higher complication rates, including perforation and bleeding [1].

## 5 Discussion

The esophagus is frequently a target for infections in immunocompromised individuals, particularly transplant recipients, those receiving chemotherapy or steroids, and organ transplant recipients. However, concurrent infections by multiple viruses are rare [2, 3].

Various microorganisms, most notably Candida, HSV, and CMV, can cause infectious esophagitis. HSV‐related esophagitis primarily affects immunocompromised hosts, although it can also occur less frequently in immunocompetent individuals [4]. Endoscopically, characteristic findings include erosions and distinct ulcers with yellow borders, predominantly in the distal esophagus [5, 6]. Microscopically, these ulcers exhibit multinucleated squamous cells at their edges, displaying nuclear molding and a ground‐glass appearance with chromatin margination and eosinophilic intranuclear inclusions (Cowdry A‐type) [6].

CMV esophagitis also tends to occur in immunocompromised patients such as those with HIV, long‐term steroid users, and organ transplant recipients. The ulcers associated with CMV are larger and differ from those caused by HSV, being linear, longitudinal, deep, and typically located in the distal esophagus [5, 6]. Histologically, these ulcers demonstrate viral cytopathic effects at the ulcer base in infected endothelial, stromal, or glandular epithelial cells characterized by cellular and nuclear enlargement and large intranuclear inclusions separated from the nuclear membrane by a halo. Cytoplasmic inclusions may also be observed [6].

Bonacini et al. and Wilcox et al. investigated the causes of esophageal infections in patients with HIV, identifying CMV as a frequent cause, followed by idiopathic ulceration and infections by HSV, with coinfections of CMV and HSV being particularly rare [3, 5]. Further investigations in transplant recipients by McDonald et al. demonstrated that a significant proportion develop infectious esophagitis, primarily caused by HSV, CMV, and Candida, with a notable incidence of coinfection between CMV and HSV [7, 8].

Our case highlights the clinical complexity and diagnostic challenge of esophagitis due to concurrent CMV and HSV‐1 infections in a post‐liver transplant patient. While single CMV and HSV infections are relatively common in post‐transplant patients due to their immunocompromised status, simultaneous infection of the esophagus with both viruses is notably rare but critical to recognize due to potentially severe complications [3]. While the number of reported cases remains scarce, a recent series by Bannoura et al. shed light on the coinfection of the esophagus with CMV and HSV in immunocompromised patients [9]. However, none of these cases exhibited such a severe and distinctive gross endoscopic presentation as observed in our patient's case.

## Author Contributions

**Ammad Javaid Chaudhary:** conceptualization, data curation, formal analysis, methodology, project administration, writing – original draft. **Taher Jamali:** conceptualization, investigation, methodology, project administration, writing – original draft. **Abdullah Sohail:** conceptualization, methodology, project administration, writing – original draft. **Christian E. Keller:** visualization, writing – review and editing. **Allyce Caines:** conceptualization, data curation, project administration, writing – original draft. **Mazen ELatrache:** conceptualization, supervision, validation, writing – review and editing.

## Consent

Written informed consent was obtained from the patient to publish this report in accordance with the journal's patient consent policy.

## Conflicts of Interest

The authors declare no conflicts of interest.

## Prior Presentation of the Case Report at a Professional Meeting

None.

## Data Availability

Data are available upon request from the authors.
